# I Walk My Dog Because It Makes Me Happy: A Qualitative Study to Understand Why Dogs Motivate Walking and Improved Health

**DOI:** 10.3390/ijerph14080936

**Published:** 2017-08-19

**Authors:** Carri Westgarth, Robert M. Christley, Garry Marvin, Elizabeth Perkins

**Affiliations:** 1 Department of Epidemiology and Population Health, Institute of Infection and Global Health, University of Liverpool, Liverpool L69 7ZX, UK; robc@liverpool.ac.uk; 2 Institute of Veterinary Science, University of Liverpool, Liverpool L69 7ZX, UK; 3 Department of Life Sciences, University of Roehampton, London SW15 5PJ, UK; g.marvin@roehampton.ac.uk; 4 Department of Health Services Research, Institute of Psychology, Health and Society, University of Liverpool, Liverpool L69 7ZX, UK; e.perkins@liverpool.ac.uk

**Keywords:** animals, dogs, exercise, happiness, health behaviour, human-animal interaction, physical activity, qualitative research, walking

## Abstract

Dog walking is a popular everyday physical activity. Dog owners are generally more active than non-owners, but some rarely walk with their dog. The strength of the dog–owner relationship is known to be correlated with dog walking, and this qualitative study investigates why. Twenty-six interviews were combined with autoethnography of dog walking experiences. Dog walking was constructed as “for the dog”, however, owners represented their dog’s needs in a way which aligned with their own. Central to the construction of need was perceptions of dog personality and behaviour. Owners reported deriving positive outcomes from dog walking, most notably, feelings of “happiness”, but these were “contingent” on the perception that their dogs were enjoying the experience. Owner physical activity and social interaction were secondary bonuses but rarely motivating. Perceptions and beliefs of owners about dog walking were continually negotiated, depending on how the needs of the owner and dog were constructed at that time. Complex social interactions with the “significant other” of a pet can strongly motivate human health behaviour. Potential interventions to promote dog walking need to account for this complexity and the effect of the dog-owner relationship on owner mental wellbeing.

## 1. Introduction

Low levels of physical activity are associated with health issues such as obesity, chronic diseases [1] and poor mental health [2]. Social systems are also important for human health and wellbeing [3]. Walking with a dog is the most common reason for visits to natural environments in England [4] and dog walking is a recognised potential mechanism for increasing physical activity [5,6], social interaction [7,8] and social capital [9]. Lack of exercise is also associated with obesity in dogs [10], providing dual benefits of dog walking for human and animal welfare. At the population level, dog owners are more physically active than people without dogs [11]. Dogs are a unique motivator for sustained physical activity despite psychological and practical barriers such as bad weather [12,13]. However, the distinctive nature of walking with a dog is poorly understood [14]. 

An owner briskly walking their dog for at least 30 min each day easily exceeds the 150 min recommended as minimum duration of moderate physical activity per week [1]. If all dog owners did this it would dramatically boost population levels of physical activity. For promotion of dog walking to be an effective intervention to improve owner health it is essential to understand what motivates dog owners to do it, as not everyone walks with their dog regularly [11]. This knowledge may also enlighten other successful ways to promote exercise. 

Animals are becoming recognised as legitimate subjects of sociological enquiry [15]. Questionnaire survey data suggests that the strength of the dog-owner relationship has both a strong association with dog walking behaviour and a large effect size [16], and has been attributed to concepts such as: attachment [17,18]; social support [19]; motivation [19]; obligation [20,21]; encouragement [21,22]; and “knowing dog enjoys going for a walk” [19]. These constructs have been shown to be primary factors associated with dog walking behaviour, but it remains unclear how these factors operate. 

Motivation for dog walking has been framed almost exclusively in terms of the needs of the dog [23]. Elderly people participating in a loaned dog walking programme reported that the dogs “need us to walk them” [24] and most dog owners report that exercising their dog regularly is good for the animal’s health [25]. Pilot intervention studies targeting the canine need for exercise, rather than the human’s, have had some success in increasing owner activity [26]. However, a recent study suggests that intrinsic motivators (e.g., finding an activity pleasurable) seem to be more important with regard to dog walking than extrinsic motivators (for the purpose of a reward outside the activity itself, such as reducing feelings of guilt) [14]. The perceived energy level of the dog [14], size [14,22], and breed [27] are also considerations. 

The way in which interactions between humans and dogs affect motivation to walk are complex and hitherto little researched or understood [14]. Qualitative research methods designed to understand social phenomena, and how people make sense of their social world [28], are ideal for studying this complexity. This study was designed to explore the perceptions, interpretations and experiences of different dog owners regarding owning and walking their dog(s), using interviews and observations. In particular, we wanted to understand how people conceptualise dog walking, what motivates and de-motivates them, and how these beliefs and perceptions influence dog walking behaviour. The findings elucidate how social relationships, including non-human, can influence human health behaviour.

## 2. Materials and Methods

In-depth semi-structured interviews (see end of manuscript for interview schedule (Appendix A)) were conducted with members of 12 dog owning households. Households were located mainly in the North-West UK and were recruited through advertisements on social media, in shops and community centres, and through word-of-mouth. Applicants were purposefully sampled in order to include regular and infrequent dog walkers, families with children, and a variety of dog types. Interviews (approx. 2 h) were conducted in the owner’s home. In addition, the researcher accompanied participants on a “typical” dog walk if the dog was walked. 

In addition, 14 short interviews (10–20 min) were conducted: ten were of dog owners walking their dogs in one of two Liverpool parks; and four at a dog show with owners of large breeds associated with high levels of exercise (Foxhound and Old English Sheepdog) and low levels of exercise (Afghan Hound and Pyrenean Mountain Dog) [27].

In total 38 people were interviewed (excluding very young children, See Table 1). All interviews were conducted by the first author, except four short interviews of owners walking their dogs, which were conducted by the second author. Data were supplemented by autoethnography of the first author’s dog walking experiences which were recorded and reflected on over a two year period. 

Participant interviews (including conversations that took place on the walks) were recorded and transcribed. A grounded theory approach was used, based on the idea that “knowledge” is constructed and embedded in human perception and social experience and that issues are individually experienced and rooted within agreed social norms or standards [29]. True to a grounded theory perspective, an inductive approach was used, drawing on wider theories as deemed appropriate for the themes that emerge from the data, rather than pre-defining a theoretical perspective. Data collection and analysis overlapped where practically possible. Primary line-by-line open coding of transcripts and diaries was conducted by Carri Westgarth assisted by Elizabeth Perkins, and axial and selective coding emerged collaboratively during discussions. Coding was managed in NVIVO software (QSR International, London, UK). As the data were coded, similarities and differences across the data were explored until theoretical saturation in emergent categories was reached. Regular discussions with the other authors assisted in critical analysis. The use of the primary author’s own dog walking experiences required a critical reflexive approach in which the emerging data provoked and challenged thoughts and feelings about dog walking and led to some of the most critical insights in the study. The study was conducted in accordance with the Declaration of Helsinki, and the protocol was approved by the University of Liverpool Veterinary Ethics Committee (Project code VREC121). Formal interview participants provided full informed written consent. Participants interviewed at the park and dog show provided audio recorded verbal consent after receiving an explanation about the purpose of the study. All interview participants were provided with a written information sheet with contact details should they wish to contact the researchers for any reason. Names of people and dogs have been changed to maximise anonymity [30].

## 3. Results

**Figure 1:**
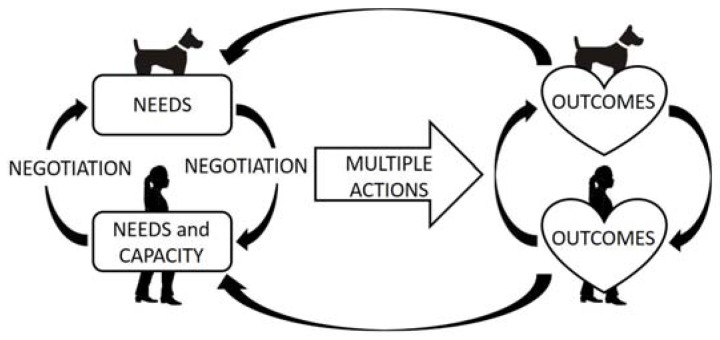
Model of dog walking negotiation. Schematic representation of interplay between the construction of dog and owner needs (which include internal and external influences; for example, enculturation), multiple possible actions taken by the owner, and perceived outcomes for dog and owner (internally and externally; for example, connectedness).

Analysis suggests a complex inter-relationship between the dog’s and the owner’s needs (see Figure 1). Participants identified needs of their dog that they aimed to fulfil through walking their dog. They also reported positive outcomes that they believed the dog gained through being walked. However, the owners’ needs were threaded through the dog’s needs; such that meeting the dog’s needs produced positive outcomes for the dog owner, the primary focus of discussion. The interplay of beliefs and perceptions is dynamic and is constantly adjusted and renegotiated as circumstances and needs change. What is perceived to “fit” with that particular dog and its owners is uniquely constructed and may vary over time both on a daily and weekly basis as well as in the longer term. The complex interplay of beliefs and perceptions based on the needs of the dog-owner dyad resulted in a range of actions, with dog walking being just one component. Normative “rules” regarding what is responsible behaviour in relation to dog walking and the “right” way to own dogs, underpinned the owner’s accounts. 

### 3.1. Construction of Dog Needs

#### 3.1.1. A Fundamental Need for Exercise

Owners described walking as being principally done “*for the dog*”. Exercise was universally expressed as a fundamental need of the dog and walking a “*responsibility*” of dog ownership. This need was largely based anthropomorphically on the relationship between physical and mental health and exercise in human beings.

“The best way to put it is, see him as yourself and if you get fat you don’t like it and he won’t like it. Maybe he’ll go on a downer, I don’t know, but I’m trying to keep him fit and healthy and treat him like I am myself (…), I try and put myself in his shoes and if it’s something which clearly I don’t like, for instance trapped in four walls all day, that’s torture and I can’t see that being a nice thing.”

—Adam

“Because they need to be fit, they can’t just be lumpy in fatness and they can’t laze around because it wouldn’t be good for them.”

—Child

“Walking” was the primary form of exercise required but other activities were playing with a ball, running with a bike, agility or games. 

Doing what’s “*best for the dog*” followed from having a strong relationship with the dog:

“If you’ve got a strong relationship with your dog you want to do what’s best for them.”

—Mary

Walking, in particular off-leash, was a component of providing the dog with a “good life”, including in the contexts of the needs of a dog to be “free from cruelty and mistreatment”, and to “have fun”:

“He gets lots of exercise, a minimum of 2 hours a day. He gets nice food. Well, he’s never complained (laughter). Yeah he is treated well, he’s not mistreated, he’s NEVER been hit. (…) I think he has got a good life.”

—Charles

The role of motivation for dog walking, as primarily described as a feature of “caring for the dog” in terms of providing exercise, socialisation and toilet breaks, identifies with previous research [23].

#### 3.1.2. Normative Guidelines on Dog Walking

Daily exercise was identified as a suitable minimum general standard. However actual frequency and length of walks varied widely from three hours a day to never. Common beliefs emerged regarding the factors which influenced the amount of exercise each dog required. Old, ill or very young dogs required less. Variations in exercise requirement reflected the size and the breed of the dog; size variation explained through physical mechanics but could be overridden by beliefs about the nature of a breed:

*“Well, the size of a dog, take your (BLINDED)* (Pug X). *The amount of steps that she’s got to take per metre have got to be far greater than what Ralph* (Alaskan Malamute) *does. So I wouldn’t expect her to need as much exercise as him”.*

—Charles

*“I do accept different breeds need different things. I do know that because I know actually the really big breeds and even like the likes of Whippets don’t actually need that much, do they. Again, very small ones. I think your medium size and your working dogs are the worst for needing (…) it’s breed and temperament, isn’t it. I know I’ve got a dog that has high needs, I do know that and all the stuff on (breed) say that.”*

—Diane

##### 3.1.3. Enculturation

Memories of dog ownership through childhood often formed the basis for explaining dog walking habits in adulthood. Equally, the participants in this study also recalled the reluctance of parents to get a dog being presented in terms of a dog’s requirement to be walked daily. Participants reported their dog walking activity as unremarkable in the context of friends and family members who also walked their dogs every day. Communities of dog walkers existed who discussed dog owning practices. Other information sources were their vet, dog trainer, breeder, groomer, rescue charity, pet shop, the internet, dog magazines and television. While a daily walk was generally considered a minimum requirement, there were occasions on which the dog owner justified not walking the dog:

“I felt guilty about not taking them for a walk, but then I’d kind of look at them and go, “They don’t seem bothered by it. They’re chilling out, they’re not barking, they’re not bugging us for attention, they’re not chewing.”

—Nadine

In the absence of adverse consequences and behavioural issues resulting from personal experiences of not walking the dog it was retrospectively identified as acceptable.

##### 3.1.4. Dog Behaviour as a Motivator

The owner’s interpretation of the behaviour of the dog was paramount in justifying going for a walk, or not going for a walk. “Subjective assessments of the dog’s ability to enjoy or cope” [23] has previously been highlighted as a justification for reducing the dog’s (and in turn the owner’s) physical activity, and we explore in more detail here the connections to perceptions of old age, ill health, and fear and nervousness. Owners interpreted their animal’s behaviour in relation to exercise and reflected perceived importance of the walk to the dog:

*“Her tail’s up. Her ears are up. She looks like she’s having a great time. It makes you think that.”*

—Emily

“I would say if you want to do the right thing for your dog, make your dog happy, then take it for a walk, make it all nice and happy.”

—Child

In contrast, other dogs were described as “lazy”. For many there was a perception that the dog did not like going out in bad weather:

“He lets me know. If it’s raining, we’ll walk half way down the main path there like that and he’ll just stop and look at me to say, “…this is stupid this. Let’s get back in the car and go.” (Laughter), but he’ll just turn round and he’ll just trot up to the car.”

—Harold

Dog behaviour could also demotivate walking if it was perceived that it was not “best for the dog”, for example, in the case of fearful or nervous dogs:

“He was the main reason why we started to realise that dogs don’t always need to be walked every day. For him, it was too stressful, to go on a walk every day”

—Nadine

Observation of changes in dog behaviour were instrumental in adjusting the length and frequency of dog walks. Older, visibly stiffer dogs were reported to require less exercise whilst “active” or “working” dogs that behaviourally demanded exercise by getting excited and pestering required more. One respondent, reflecting on her dog walking, recognised that it was difficult to disentangle the dog’s desire to be walked from the way in which she might have shaped the dog’s behaviour.

“It is partly led by him, it really is. I know he is not a lap dog, he’s a working dog. He demands it and you would have seen he’s demanding me to walk in a way that I never saw (Name’s) dog do. But at the end of the day what came first? It’s one of those. Does he demand because he expects and he gets, or do I give him it and he gets it because he’s demanding it?”

—Diane

Given the deeply intertwined relationship between human and animal it is not surprising that Diane reflects the difficulties of separating the owner’s needs from the dog’s needs. 

### 3.2. Dog Outcomes

Perceived dog benefits from exercise included: increased fitness; preventing overweight; extended life; reduced veterinary fees; mental stimulation; reduced frustration, destruction and aggression; and opportunities for socialisation with other people and dogs.

### 3.3. Owner Needs—the Issue of Capacity

Intention and desire to walk their dog also had to fit with an owner’s other commitments and abilities. 

#### 3.3.1. Health

Periods of injury or long-term illness were frequently cited as the most legitimate reason why a participant would not walk with their dog. That is not to say the dog was not walked—responsibility in some cases was devolved to someone else. Individuals varied in the extent to which similar injury or illness limited their ability to walk their dog and were also affected by the availability and suitability of substitute dog walkers and relative accessibility of dog walking locations. 

#### 3.3.2. Time

Time, or lack of it, was the most commonly reported constraint on dog walking. Managing young children and their routines, or other commitments of older children such as school and clubs, had to also be considered when negotiating time for dog walking. However, not all participants were convinced that lack of time was a valid reason for not walking a dog:

“Erm.....health, is the only excuse that I can give for people not walking their dogs. I think everything else is an excuse. Beyond that. People that say that they don’t have time to walk a dog, shouldn’t have a dog. Personal opinion. (…) My dog doesn’t need it. My dog doesn’t like going out in the wet. My dog’s lazy. No it’s not, you are.”

—Alice

#### 3.3.3. Routines and Co-Discipline

Some participants reported that dogs gave them the self-discipline to get up early, go out in the dark or the bad weather, and not be easily put off from walking. However, others such as Nina were unaffected and described herself as “lazy” and “selfish”. Some owners had strict routines and rituals, walking in the exact same time and place every day, taking the same route, with the same people. Others were more flexible but still structured around regular events, such as before or after work or school. Dogs were described as “knowing the time” and becoming excited in anticipation of going for a walk, thus perhaps self-discipline here is better described as *co-discipline*. Habit formation has been shown to be useful in promoting physical activity [31] and may have an important role in dog walking [32], as we confirm here. 

### 3.4. Owner Outcomes

#### 3.4.1. Owner Happiness

Our data confirms the value of the effects of dog walking on owner psychological rather than physical health [33]. Dog walking was often described by our participants as relaxing and stress-relieving. These terms were used to describe both how they felt while they were out walking their dog as well as the motivation for walking:

“With my job being quite stressful at times it is relaxing. It might not seem it when I am getting them in and out the van but when I’m actually out there and I am by myself with just the dogs it is my chill time. I’ve got to do it every day for my sanity let alone theirs”

—Samantha

Although walking in general is known to be stress-relieving, it was clear that walking for most dog owners was enhanced by the specific presence of dogs and desire for the “fun” they bring [34]:

“It just feels special when they’re there with you. It makes a good walk an excellent walk. It’s that little bit more when you’ve got an animal by the side of you.”

—Mary

“It’s not just about the physical activity they give you, it’s the mental benefits. My friend who doesn’t have her own dog comes walking with us and says that it’s impossible to leave depressed after watching the dogs running around enjoying themselves.”

—Excerpt from conversation recorded in ethnographic diary.

The positive emotions experienced by dog owners on a walk were rooted in the emotions that the dog was perceived to exhibit:

“I thoroughly enjoy it because I think he enjoys it and I love the thought of him being happy, so to know that he is out somewhere new and he is enjoying himself and that he’s allowed to sniff and he is bouncing around and you can tell that he is excited, I love that.”

—Nina

This description of “vicarious pleasure” demonstrates how dog walking can produce a shared “happiness”; the owner deriving their pleasure from the pleasure they interpret from the animal’s behaviour. Our findings explain why intrinsic motivation for dog walking is connected to the dog [33]. There were a small number of situations where walking with their dog was not pleasurable, for example where dogs were felt to be challenging on a walk either due to health or behavioural reasons. These walks often presented difficulties for the owners, but most importantly, the perception that the dog was not finding the walk pleasurable contributed to a negative owner experience.

#### 3.4.2. Owner Exercise

In contrast to dog exercise, owner exercise was reported to be a secondary bonus and not a primary motivator [33]:

“Mitch: Just to make sure that he’s healthy, his mind is... That he is doing dog things, I suppose, when he’s getting out. That’s about it. It’s nice for us to get out and exercise as well.

Interviewer: Yes. Was that part of the consideration when you got Ozzie?

Mitch: It wasn’t actually, no, but it’s just a benefit of having a dog, I suppose.”

A few owners suggested that they got a dog in order to increase their exercise. Dog walking was described as particularly valuable as a means of exercise because it “*doesn’t feel like exercise*”, as reported elsewhere [35]. Dogs also lent “legitimacy” to be out walking in green spaces; people walking alone without dogs were viewed as “odd”. Most owners felt that dog walking helped to keep them physically active and healthy, with some reporting increased physical activity, weight loss and improved management of health conditions since owning a dog. For some, dog walking was their only physical activity, whereas for others it was additional. However, dog walking had opportunity costs and was sometimes undertaken at the expense of other high intensity exercise, like going for a run or to the gym, because the dog was the priority.

#### 3.4.3. Connectedness

Dog walking brought participants into connection with nature and their surroundings, other people, and also their own dog. Walking with a dog encouraged social contact with people, and dogs were recognised as a key “ice breaker”. Conversations were usually fleeting without revealing personal information [30,34,36], but occasionally acquaintances developed into firm friendships. However, the significance of these brief interactions, and the power of the dog as integral to the relationship, are not to be underestimated, for example, a participant who regularly met the primary author on dog walks was visibly upset by the death of the author’s dog, both at the time and again in later interviews. 

Although enhanced social contact was reported as an outcome of dog walking, it was only perceived as a need in specific circumstances, for example an elderly gentleman living alone. Some owners were more interested in the solitary experience of being with their dog, and saw the interactions with other people and dogs as unwanted:

“We don’t get much time for us; it’s quite nice me time so, even though I’m out with the dog and we’re doing whatever, it’s nice to be alone with your thoughts, just to sort of relax and think more than anything else. So when someone else’s dog comes up and bursts your bubble, (Laughter) it literally goes pop and the whole world comes down; it’s like, “Oh for God’s sake.”

—Jake

The human social interaction that is promoted through walking with a dog is well-documented [7,8,9], but it was previously not known if this is motivating [5]. This study clarifies that, social interaction with people is not a prime motivator except in specific circumstances. In fact, the interruption caused by social interaction may diminish the value of dog walking as a stress-relieving activity. Dog-owners also reported that the act of walking strengthened their relationship and connectedness with their dog, in turn motivating more walking. One participant described the relationship with his dog through walking as “*symbiotic*”:

“…one thing lives off another and it gets better”

—Barry

However, others suggested that walking did not always have to be integral to their relationship with the dog:

“I feel like I can compensate and if you like I still maintain that relationship with him. I don’t feel like he loves me for the fact that I take him for a walk, although seeing him with mum I know that it’s a big influence and factor that he does. I think he just adores her and I love that, I love it, but my relationship with him I don’t think is based on walks (…) I think for me I just smother him in love physically and emotionally and that’s how we maintain our relationship.”

—Nina

Our participants talked at length about their interactions with their dogs including; cuddles on the sofa, watching them “do funny things”, or feeling rewarded from seeing improvements in behaviour of a dog that they had “rescued” and rehabilitated. Like previous research, walking was identified as just one component of the emotional benefits of a human-animal relationship [37].

### 3.5. Negotiating Needs

Owner and dog needs arose in discourse as separate and distinct from each other, but in their presentation they appeared to match. When examining participant accounts it became clear that this congruence was often constructed. The nature of the exercise provided to the dog varied according to the needs and capacity of the owner but was presented in a way that justified the amount, when or where they walked their dog, in terms of the needs of the dog. For example, an illustration from the ethnographic diary demonstrates how owners use a human-dog need negotiation process in order to reach a point of constructed compromise:

“I was working from home and the day flew by, and it was early afternoon and I still hadn’t had time to take the dogs out. I found myself thinking about someone I had recently interviewed and how they justified giving the dogs a bit of play time instead of a walk, if they don’t have time to take them out. So I took (BLINDED) out in the garden and played fetch for a few minutes. (BLINDED) looked tired out so I let her sleep. I thought “she’s getting old now and will be ok without a walk today, I will give her a rest”. (…)

As I was throwing the ball again for (BLINDED) in the garden I suddenly thought WHAT AM I DOING! It was like a wake-up moment....I really shouldn’t not walk them. This project makes me feel hugely guilty when I notice myself constructing justifications as to why I don’t need to walk them, because it fits with my own needs that day.

So I put their leads on and took them out. Sod work.

Although we only went around the block. Better than nothing but I was really running late!”

—Ethnographic diary

In summary, the principle of exercising the dog daily provided an overarching framework within which owners adjusted the practice of the dog walk according to a host of human, dog and environmental variables. Understanding the complexity of whether, when, and for how long an owner exercises with their dog has important implications for any strategy which seeks to promote dog walking as a public health intervention. Our findings also contribute to understanding how perceived responsibilities towards “significant others” can influence human physical activity behaviour.

## 4. Discussion

This paper has provided a detailed understanding of why dog ownership can be such a strong motivator for sustained physical activity. The primary reported motivation for dog walking was the perception of the dog’s need for exercise. In contrast, however, the primary valued outcome was that of increasing owner’s mental wellbeing through providing a pleasurable and stress-relieving experience. Human physical activity, although beneficial, was a secondary outcome. Perceived responsibility to walk a dog depended primarily on the perceived needs of the individual dog at that time (what was “best for the dog”), but also the perceived needs of the owner and the owner’s ability to meet the dog’s needs within the compromise. Personal views of what a dog owner is expected to do with regards to walking their dog provide a framework in which decisions are made and which vary depending on the owner’s social circles and historical and personal contexts. 

Social support from an important “other” can have positive effects on motivation for physical activity, for example family and friends [38,39,40]. This study elucidates how this can also happen with a pet dog, and why they are particularly motivating. The dog-owner relationship underpins our findings and elucidates why constructs relating to this have previously been found to be strongly associated with dog walking [16]. Similar to findings by Sanders, pet dogs were viewed as conscious beings and able to communicate intentions and emotions; owners routinely used their day-to-day experiences with their pet dogs in order to understand them as socially defined “persons” [41]. Our participants agree with the argument that animals can experience happiness [42] and that we, as humans in close relationships with companion animals, are able to perform an interpretation of animal behaviour in a similar manner to that we would do with other humans [43]. We find that motivation for dog walking is provided through the significant other who is not only sharing the pleasurable experience, but is fundamental to producing it. The dog in dog walking is central to making us happy; the benefits of dog walking are not just from being in nature or being a conduit to exercise.

Drawing on theoretical and practical evidence regarding other human relationships may be helpful in exploring how owners described a sense of responsibility towards their dogs. Rather than there being hard and fast rules about how we “ought” to behave in regard to obligations towards our kin, we use normative guidelines to engage in a process of actively working out what to do in that particular context [44]. Likewise, here we have discovered a number of considerations to be made when an owner negotiates their responsibility to walk their dog, but no clear rules as to what they must eventually do. These socially constructed guidelines appear to be well recognised (and also echoed in other studies e.g., [45]), however, each owner may come to a different conclusion as to what the final action should be, and this will vary across contexts and dogs. Thus, in practice, the frequency and intensity with which people exercise their dogs varies widely. 

Interestingly we did not note gender differences as observed with obligations regarding human kin relationships [46]. This requires further exploration, however, we may tentatively hypothesise that gender differences in how care is given may be attenuated in this context, as dogs may be seen as a legitimate vehicles for males to show affectionate care-giving behaviour; it is still “manly” to walk and show affection to one’s dog. This is supported by a study that showed no differences in attachment, play behaviours or physical comfort given to dogs by male or female owners [47]. 

To most participants, walks were a general principle to be followed, however others used other forms of dog “exercise” legitimately. This has implications for motivating owner physical activity: (i) some will not be motivated by efforts to make them walk if they can justify other forms of exercise for the dog; and (ii) the human “exercise” motivated could be not just walks, but games for the human to play with their dog. This has been a missed opportunity so far in intervention strategies (although was promoted with children in [48]). Participants also suggested more education around dog needs was required if dog walking was to be promoted, however, nobody reported perceiving that dogs did not need walking; increased knowledge does not make people’s behaviour change [49].

Sharing key rituals with animals is interesting given the view of shared rituals as a source of social cohesion [41]. Strengthening of the dog-human bond through routines may explain why a dog is considered such a unique source of social support for walking. The role for routines and habit development in interventions to promote dog walking requires testing [5], in particular as our findings show how dog behaviour through “pestering” and excitement can be integral to motivation. Even if we accept that some dogs have personalities and energy levels more conducive to this effect (perhaps to be carefully targeted upon dog acquisition), there is also scope for modification of individual behaviour through simple training techniques using positive reinforcement. 

This study also supports the notion that intrinsic motivation is paramount in dog walking [14]. Interventions could target the perception of dog happiness and wellbeing through dog walking, and thus owner happiness. In particular, these need to be directed towards older, smaller, or perceived “lazier” breeds of dogs, for which explicit motivation through the dog’s direct behaviour is likely to be minimal, and socially constructed barriers around perception of their need for walks require addressing. 

Our study may explain why pilot dog walking interventions have not been particularly successful, for example using canine health messaging [26] or social networking [50]. Regarding the latter, social interaction is not an important motivator for dog walking. Owners are constantly negotiating and renegotiating their responsibilities towards walking their dogs based on complex constructed needs of both themselves and another (their dog) at that point in time, and interventions may not translate into behaviour change as readily as one might hope. Owners are torn between responsibilities not only to their dog but to their other family members, friends and work, among others. Despite a logical health benefit for both themselves and their dog, it may not make sense within their own lives to adopt a new strategy. Any proposed interventions to increase dog walking must be sympathetic to this and take a holistic approach, so that if one reason for not walking with the dog is addressed, it is not simply replaced with another. For this reason, multi-level interventions addressing a variety of factors conducive to dog walking are likely to be the most successful [5]. As suggested above, these could include: routine and habit development, including training dog behaviours which support this; promotion of mental health benefits and “shared happiness” for both owners and dogs; expanding other ways to “enjoy exercise” together, such as training tricks and playing games; addressing perceptions about exercise needs of smaller “less active” breeds; and overcoming perceived human health barriers both at an individual level and through the provision of improved local environments in terms of physical accessibility for dog walking.

This study has a number of strengths compared to previous research, which often used focus groups [45,51,52], small sample sizes [23,33,37,52], convenience samples [30,33], predominantly female subjects [30,33,37,45,51,52,53], only one individual from each study household [23,33,37,45,51], and only people who already walked their dog regularly [30,33,36,51,52,54,55]. Data was also obtained during a dog walk with the participants, as opposed to just talking about these walks [33]. Thus, we feel that it is the most in-depth study of dog owner’s beliefs and perceptions relating to dog walking to date, and this is reflected in the complexity and novelty of our findings. In line with best practice in qualitative research, the robustness of the study findings were maximised by the critical reflexive approach used, collaborative discussions, a rigorous process of coding and collecting the data in line with the constant comparative method, and triangulation of sources (interview, observation and autoethnographical diaries). Further, the use of purposeful sampling techniques and the reaching of data saturation lend further credibility to our findings.

The study has a small number of limitations. As with all studies, the sample comprised people who were willing to participate and in this case involved people who were willing to talk about their dog walking activities. Although some of the participants reported rarely walking them, it is possible that a larger sample would reveal an even greater variation in dog walking than that elicited in this study. However, even participants who walked their dog regularly could describe instances where they chose not to. Notwithstanding the small sample size, participants in this study identified complex and hugely varying patterns in their attitudes to and practice of dog walking. Data collection was carried out primarily in the North-West UK with mainly white ethnicities. It would be valuable to conduct studies with greater ethnic and cultural diversity in different geographical contexts. However, the agreement with research from other countries leads us to believe that it is a representation of at least the lives of some typical pet dog owners. In addition, future studies might also explore the role of exercise in dogs kept for a more utilitarian purpose such as dog racing, shepherding, guarding, and so on. The cultural and domestic context within which this study took place may also limit the transferability of these findings to dog owners in other countries, in particular, in countries where pet owners have access to large back yards such as in parts of the US and Australia. 

## 5. Conclusions

In conclusion, social relationships, even with non-human others, can impact physical activity behaviour, through engendering a sense of responsibility to another and shared pleasure. Dog walking is used to meet the emotional needs of the owner as well as the physical needs of the dog. Possible key points for future intervention to increase dog walking are to promote how it may increase the dog’s, and thus the owner’s, happiness, or targeted habit formation. However, behaviour change is unlikely without addressing the needs and perceptions of the owner about both themselves and the dogs, on an individual and ongoing basis. 

## Figures and Tables

**Table 1:** Participant information of dog owner interviews about dog walking.

Interview Type	Gender(s)	Ethnicity(s)	Age(s)	Occupation(s)	Dog(s)	Frequency Dog Walked	Walk Observed
Full	F	White	51	Associate professional and technical	MN Poodle/spaniel 10 years, FN Border Terrier 10 years	Twice daily	No
Full	M	Mixed	62	Retired	MN Alaskan Malamute 5 years	Twice daily	Yes
	F not present	White	49	Skilled trade			
Full	M	White	69	Retired	ME Labrador 4 years	Twice daily	Yes
	F not present	Unknown	Unknown	Retired			
Full	F	White	36	Student	ME Spanish Water Dog	Three times daily	Yes
	Child M		2	Child			
Full	F	White	42	Associate professional and technical Associate professional and technical children	FN Border collie/Springer spaniel	Once–twice daily	Yes
	M		45				
	Child M		10				
	Child F		5				
Full	F	White	38	Professional	FN Labrador 9 years, ME French Bulldog 1 year, FE French Bulldog 8months	Once–twice daily	Yes
	Adult M not present		52	Manager			
	Child F		9	Children			
	Child M		7				
Full	F	White	68	Retired	MN Border Collie 12 years, ME Border Collie 7 years	Never	No
Full	F	White	58	Manager	MN Cavalier King Charles Spaniel 3 years, plus regular visiting MN Cavalier King Charles Spaniel	Daily	Yes
	M part-present		56	Manager			
	F		28	Professional			
Full	F	White	52	Permanently sick or disabled	MN Nova Scotia Duck Tolling Retriever 14 years	Several times a month	No
Full	F	White	29	Professional	MN Husky/Malamute 7 years, FN Labrador 4 years	Several times a week	Yes
	M		29	Professional			
	Child M		2	Child			
Full	F	White	63	Retired	MN Old English Sheepdog 9 years, MN Old English Sheepdog 7 years	Once–twice daily, short lead walk	Yes
	M		68	Retired			
Full	M	White	40	Manager	FN Jack Russell 7 years, MN Cocker Spaniel 4 years	Daily	Yes
	F		44	Manager			
Mini-dog show	M	White	Adult	Unknown	Four Old English Sheepdogs	Twice daily	No
Mini-dog show	M	White	Adult	Unknown	Afghan Hounds	Intermittently	No
Mini-dog show	F	White	Adult	Professional	Eight foxhounds and two Border Collies	Daily	No
Mini-dog show	M	White	Adult	Unknown	Pyrenean Mountain Dog	Daily	No
Mini-park	M	White-Asian	Adult	Unknown	M Jack Russell 10months	Daily	Met on a walk
Mini-park	M	White	Adult	Unknown	F Staffordshire bull terrier, unknown age	Three times daily	Met on a walk
Mini-park	M	White European	Adult	Elementary	M American Staffordshire Bull Terrier *, M American Staffordshire Bull Terrier */Labrador	At least daily	Met on a walk
Mini-street	M	White	Elderly	Retired	M Labrador 13 years	Daily, short lead walk	Met on a walk
Mini-park	M	White	Young adults	Unknown	M Pug 6months	Daily	Met on a walk
	F						
Mini-park	F	White	Adults and children	Unknown	F Chihuahua 5 months	Daily	Met on a walk
	M						
	plus 3 children playing						
Mini-park	M	White	Adult	Unknown	M Rottweiler, F other dog	Daily	Met on a walk
Mini-park	M	White	Adult	Unknown	M Jack Russell Terrier/Yorkshire Terrier 13 years	Daily	Met on a walk
Mini-park	F	White	Adult	Unknown	M Jack Russel 4 years, F Jack Russell 3years	Three times daily	Met on a walk
Mini-park	M	White	Adult	Unknown	M King Charles Spaniel	Twice–three times daily	Met on a walk

M = Male, F = Female, E = Entire, N = Neutered. *: American Staffordshire Bull Terrier is a pseudonym for Pit Bull Terrier Type in this geographical area.

## Interview Schedule

Introductory question:

Tell me about your dog(s)....

- Type
- Where did you get it?
- Why did you get it?
- Personality—what are your dog’s favourite things?
- What do you feed your dog?—main diet, treats

Key topics to cover:

- Relationship with dog(s)
  ○ What do you like about owning a dog?
  ○ Does owning a dog ever cause problems or difficulties?
  ○ What are the most/least enjoyable parts of owning a dog?
  ○ How would you describe your relationship with your dog?
  ○ Does your dog live outside or inside?
  ○ Who owns the dog?
  ○ Who is responsible for dog duties such as feeding, grooming, exercise?
  ○ Would you consider your dog to be part of the family?
- Going out with the dog(s)
  ○ Can you describe a typical dog walk?
    ▪ Who does it?
    ▪ When, how often?
    ▪ Where?
    ▪ What happens?—activity types
    ▪ Do you walk your dog on or off lead?
    ▪ How does your dog typically behave?
    ▪ How do you feel when you are walking your dog?
    ▪ What do you think about whilst walking your dog?
  ○ How close do you live to walking areas and what are they like?
    ▪ How suitable are they for dog walking?
    ▪ Which walking areas do you use (distance over suitability?). What do you look for when choosing a place to walk your dog?
    ▪ In the past where you have lived how close were you to dog walking areas compared to now? Do you think this had an effect on your dog walking when you moved house?
  ○ Do you see any other people when walking your dog?
    ▪ Can you tell me some more about the kinds of conversations you have with other people when you out walking with your dog? (Do they involve anything more than just a “Hi”?)
    ▪ Do you ever meet up with other people to go for a walk?
  ○ What do you look forward to about walking your dog?
  ○ What things don’t you look forward to about walking your dog?
  ○ Do you ever have any problems or issues when out for a walk with your dog(s)?
  ○ Can you describe any solutions to these problems that you use? Management strategies?
  ○ What sorts of things stop you from going for a walk with your dog/s?
  ○ Tell me about a time you were unable to take your dog for a walk—what happened, why did it happen?
  ○ Do you do other physical activities? How do you decide whether to walk the dogs or do other activities?
  ○ Can you describe things that you feel are motivators to walking your dog?
  ○ Can you describe any barriers to walking with your dog?
  ○ Is it much different going for a walk with a dog compared to a friend?
  ○ What sorts of benefits, if any, do you see to walking WITH your dog? As opposed to walking on your own.
  ○ You have explained a lot about your relationship with your dog, and about walking your dog—could you reflect on whether you think these two things are linked?
- Personal beliefs/values about dog ownership/walking
  ○ What do you think might prevent or make it difficult for other dog owners to walk their dogs.
  ○ If you had a friend and you wanted to encourage them to start walking their dogs, or walking with you and your dog, what would you tell them?
  ○ How important do you think exercise is for dogs? How often do they need walking?
    ▪ Where do you think this opinion comes from? Why do you believe that?
  ○ Breed of dog—how much exercise does this breed need? Does that influence how you walk your dog?
  ○ Are you think kind of person who finds it difficult or easy to get things done? How disciplined are you?
- Significant others—subjective norms—explore views of people mentioned in above (i.e., does your partner feel the same, other family members? What does your vet think?—interjections as required from above)
  ○ Who would you seek advice from about dogs? Whose opinion do you value when it comes to dogs?
  ○ Do any of your friends have dogs? Family?

**In Summary:** What would you recommend the NHS (National Health Service) as an organisation, to do if it wanted to encourage dog owners to do more walking with their dogs?

Summarise by coming up with a list of ways to encourage people to walk more WITH their dogs (Scribe onto paper). Sum up and review the attitudes and feelings shared. Validate perceptions and attitudes that have emerged.
